# 

**Published:** 1889-01

**Authors:** 


| | PAGE |
|---|---|
| RICKETS IN MONKEYS, LIONS, BEARS AND BIRDS. J. Bland Sutton, F. R. C. S. | 1 |
| NOTES ON HORNED MAMMALS, WITH SOME OBSERVATIONS UPON POLICERATE OR MULTIPLE-HORNED SHEEP. R. W. Shufeldt, M. D., C. M. Z. S. | 30 |
| SOME BRIEF NOTES ON MEDICINES USED IN VETERINARY PRACTICE. Kenelm Winslow, B. A. S., M. D.V. | 38 |
| PENTASTOMA MONILIFORME, A RARE PARASITE, WITH REMARKS ON THE PENTASTOMIDÆ. William S. Gottheil, M. D. | 42 |
| RABIES. Rush Shippen Huidekoper, M. D., Vet. | 46 |
| THE MUSCULAR COATS OF THE ŒSOPHAGUS OF THE DOMESTICATED ANIMALS. Leonard Pearson, B. S. | 59 |
| BURSATTE. John R. Anderson. | 73 |
| THE AMERICAN VETERINARY COLLEGE. | 77 |
| EDITORIAL DEPARTMENT. | 81 |
| CASE DEPARTMENT. | 87 |
| PROCEEDINGS OF VETERINARY SOCIETIES. | 93 |
| REVIEW DEPARTMENT. | 97 |
| BOOKS AND PAMPHLETS RECEIVED, | 102 |

